# Deficient leptin receptor signaling in T cells of human SLE

**DOI:** 10.3389/fimmu.2023.1157731

**Published:** 2023-03-17

**Authors:** Ting Liu, Ming Zheng, Li Jia, Mingyuan Wang, Longhai Tang, Zhenke Wen, Miaojia Zhang, Fenghong Yuan

**Affiliations:** ^1^ Department of Rheumatology, the Affiliated Wuxi People’s Hospital of Nanjing Medical University, Wuxi People’s Hospital, Wuxi Medical Center, Nanjing Medical University, Wuxi, China; ^2^ Department of Rheumatology, The First Affiliated Hospital of Nanjing Medical University, Nanjing, China; ^3^ Jiangsu Key Laboratory of Infection and Immunity, Institutes of Biology and Medical Sciences, Soochow University, Suzhou, China; ^4^ Department of Research Center, Suzhou Blood Center, Suzhou, China

**Keywords:** SLE, T cell, leptin, leptin receptor, AMPK

## Abstract

**Background:**

Systemic lupus erythematosus (SLE) is a prototypic autoimmune disease mainly mediated by IgG autoantibody. While follicular helper T (Tfh) cells are crucial for supporting IgG autoantibody generation in human SLE, underlying mechanisms for Tfh cell mal-differentiation remain unclear.

**Methods:**

In total, 129 SLE patients and 37 healthy donors were recruited for this study. Circulating leptin was determined by ELISA from patients with SLE and healthy individuals. CD4 T cells isolated from SLE patients and healthy donors were activated with anti-CD3/CD28 beads under cytokine-unbiased conditions in the presence or absence of recombinant leptin protein, followed by detection for Tfh cell differentiation by quantifying intracellular transcription factor Bcl-6 and cytokine IL-21. AMPK activation was assessed by analyzing phosphor-AMPK using phosflow cytometry and immunoblots. Leptin receptor expression was determined using flow cytometry and its overexpression was achieved by transfection with an expression vector. Humanized SLE chimeras were induced by injecting patients’ immune cells into immune-deficient NSG mice and used for translational studies.

**Results:**

Circulating leptin was elevated in patients with SLE, inversely associated with disease activity. In healthy individuals, leptin efficiently inhibited Tfh cell differentiation through inducing AMPK activation. Meanwhile, leptin receptor deficiency was a feature of CD4 T cells in SLE patients, impairing the inhibitory effect of leptin on the differentiation of Tfh cells. As a result, we observed the coexistence of high circulating leptin and increased Tfh cell frequencies in SLE patients. Accordingly, overexpression of leptin receptor in SLE CD4 T cells abrogated Tfh cell mal-differentiation and IgG anti-dsDNA generation in humanized lupus chimeras.

**Conclusion:**

Leptin receptor deficiency blocks the inhibitory effect of leptin on SLE Tfh cell differentiation, serving as a promising therapeutic target for lupus management.

## Introduction

Systemic lupus erythematosus (SLE) is a chronic multisystem autoimmune disease with an increasing disease prevalence rate, while clinical treatment options are limited (1). In clinical practice, SLE patients are dominantly young females and characterized by the production of high levels of IgG anti-nuclear autoantibodies (2). Typically, the generation of IgG antibodies requires the involvement of follicular helper T (Tfh) cells, which support germinal center reactions for somatic hyper-mutation and affinity-maturation in B cells (2–4). Therefore, molecular mechanisms underlying the differentiation of Tfh cells in SLE patients are relevant for developing therapeutic strategies.

Accumulating studies have pinpointed metabolic adaptions as the determinant for T cell differentiation and function (5–7). Specifically, AMP-activated protein kinase (AMPK), the master energy sensor that responds to increased AMP/ATP ratios and drives catabolic metabolism, is essential for the differentiation of regulatory T cells and blocks the differentiation of effector T cells including Th1 and Th17 cells (8, 9). In contrast, the mechanistic target of rapamycin (mTOR) promotes anabolic metabolism and the differentiation of effector T cells (6, 10). While mTORC1 is required for the differentiation of Th1 and Th17 cells, mTORC2 is crucial for Th2 cell differentiation (11, 12). Rapamycin, a relatively selective inhibitor of mTORC1, is known in facilitating the differentiation of regulatory T cells (13). Mechanistically, AMPK and mTOR are both activated on lysosomes, interacting for metabolic homeostasis (12, 14). Upon activation, AMPK phosphorylates Raptor and TSC2, inhibiting the stability and activation of mTOR (12, 15, 16). Such an AMPK-mTOR axis is also involved in the differentiation of Tfh cells. As such, attenuation of AMPK signaling by ROQUIN promotes the formation of Tfh cells, assigning AMPK as an inhibitory player in Tfh cell differentiation (17). In contrast, AMPK is also indicated in promoting Bcl-6 expression in T cells and endothelial cells (18, 19), suggesting a sophisticated function of AMPK in Tfh cell biology.

Leptin is a pleiotropic hormone known to regulate a wide range of systemic functions (20). By signaling through the leptin receptor (LepR), leptin plays a critical role in energy balance and metabolic homeostasis (21). Of interest, leptin inhibits AMPK activity in the hypothalamus, inducing anorexia and causing a reduction in body weight (21). On the other hand, circulating leptin stimulates AMPK activity in the peripheral, enhancing fatty acid oxidation and reducing body weight (21). The potential function of leptin-LepR signaling in SLE has long been an active area for investigation. In murine lupus induced by pristane and New Zealand Black (NZB) × New Zealand White (NZW)F1 (NZB/W) mouse model of spontaneous SLE, leptin deficiency protects from the development of autoantibodies and renal disease (22). Vice versa, elevated leptin levels associated with disease manifestations and administration of leptin promote the development of autoantibodies and renal disease in murine SLE (22). However, leptin might exert a distinct function in human SLE. In patients with SLE, while accumulating studies have identified significantly higher leptin levels, regardless of ethnicity, sample size, data type, and matched variables (23), such higher serum leptin levels might not necessarily correlate with their disease activity (24). Further, higher frequencies of variant genotype (AA) and (A) allele of the LepR gene of SLE patients are not associated with patients’ clinical, laboratory, and radiological manifestations (24). Those studies reflect a complicated function of leptin-LepR signaling in human SLE.

In essence, the involvement of AMPK in Tfh cell differentiation and the critical activity of leptin in driving AMPK activation in the peripheral raised a possible role of leptin in the mal-differentiation of Tfh cells in human SLE, which remains largely unknown.

## Materials and methods

### Patients and healthy individuals

One hundred and twenty-nine patients with SLE and thirty-seven age-matched healthy individuals were enrolled. The clinical characteristics of SLE patients were summarized in **Table 1**. SLEDAI-2000 was used to access the disease activity of clinical patients. Informed consent was obtained from all participants. Experiments were performed in compliance with the Helsinki Declaration and approved by the Institutional Review Board of Soochow University.

**Table 1:** Clinical characteristics of SLE patients.

Parameters	SLE patients	Healthy donors
Gender (n, %)	Female (129, 100%)	Female (37, 100%)
Age (years, mean±SD)	34.7±12.0	35.8±13.1
BMI (mean±SD)	22.6±4.7	–
Disease duration (months, mean±SD)	5.5±6.1	–
Manifestations		
Fever (n, %)	28, 21.7%	–
Rash (n, %)	48, 37.2%	–
Nephritis (n, %)	67, 51.9%	–
Arthritis (n, %)	25, 19.4%	–
Serositis (n, %)	21, 16.3%	–
Hematologic disorders (n, %)	43, 33.3%	–
Treatments		
Untreated (n, %)	51, 39.5%	–
Corticosteroids (n, %)	72, 55.8%	–
Antimalarials (n, %)	66, 51.2%	–
Immunosuppressants (Cyclophosphamide, mycophenolate mofetil and calcineurin inhibitors, n, %)	57, 44.2%	–

### CD4 T cell isolation and Tfh cell differentiation

CD4 T cell isolation and culture were performed as previously described (25–27). Specifically, PBMCs isolated by gradient centrifugation using Lymphocyte Separation Medium (Corning) were used for isolating CD4 T cells with Human CD4 T Cell Enrichment Kit (StemCell Technologies). The CD4 T cell purity was consistently over 90% determined by flow cytometry.

For Tfh cell differentiation, CD4 T cells were stimulated with anti-CD3/CD28 beads (cell/bead ratio = 2/1, Thermo Fisher Scientific) for 4 days, followed by analyses of linage-determining transcription factor Bcl-6 using flow cytometry as previously described (28, 29). Otherwise, cells were stimulated for 6 days, re-stimulated with Cell Activation Cocktail (with Brefeldin A, BioLegend) and analyzed for IL-21 generation. All cells were cultured in RPMI 1640 medium supplemented with 10% FBS (Thermo Fisher Scientific) plus Penicillin-Streptomycin-Glutamine (Thermo Fisher Scientific).

### Reagents

The AMPK activator A769662 and AMPK inhibitor Compound C were purchased from Sigma-Aldrich. Circulating leptin levels were determined using Leptin Human ELISA Kit from Invitrogen. Human recombinant leptin protein was from Thermo Fisher Scientific. Human LepR expression vector and the control were from OriGene, while human AMPKα shRNA and the control were from Santa Cruz Biotechnology. Human CD4 T cells were transfected with electroporation using Nucleofector kits from Lonza. All reagents were used according to the manufacturers’ instructions.

### Flow cytometry

CD4 T cells were treated with Fix Buffer I (BD Biosciences), Perm Buffer III (BD Biosciences), and stained with anti-human antibodies as follows: FITC anti-CD4 (BioLegend, Clone OKT4), PE-Cy7 anti-Bcl-6 (BioLegend, Clone 7D1), PE-anti-IL-21 (BioLegend, Clone 3A3-N2), Purified anti-AMPKα Antibody (BioLegend, Clone 5C9-5B10-6D3) plus Alexa Fluor^®^ 647 anti-mouse IgG (Thermo Fisher Scientific, Cat. A-21235), Alexa Fluor^®^ 647 mouse anti-human leptin receptor (BD Biosciences, Cat. 564376), Purified anti-AMPKα Phospho (Thr172) Antibody (BioLegend, Clone A20017A) plus Alexa Fluor^®^ 488 anti-mouse IgG (BioLegend, Clone Poly4053). Cells were stained for 45 min at 4°C. Flow cytometry was performed on an LSR II flow cytometer (BD Biosciences). Data were analyzed with FlowJo software (Tree Star Inc.).

### Immunoblots

Immunoblots were performed as previously described (30). Primary antibodies used were as follows: anti-human AMPKα (Abcam, ab187408) and anti-phosphor-AMPKα (Cell Signaling Technology, Rabbit mAb #2535). β-actin detected with anti-human β-actin (Abcam, ab8227) was used as the internal control.

### Humanized SLE chimeras

Humanized SLE chimeras representing human SLE were generated as previously described (2, 31, 32). In brief, NSG mice (Biocytogen) at the age of 8 weeks were housed in institutional pathogen-free animal facilities, and reconstituted with PBMCs (15 million/mouse) from active SLE patients through tail vein injection. NSG mice injected with the same number of healthy PBMCs were used as controls. Chimeras injected with PBMCs from the same donor were randomly assigned to control and treatment groups. Specifically, one day after immune reconstitution, chimeras were treated with human recombinant leptin protein (0.5 μg/g body weight) daily by intraperitoneal injection for 4 weeks to achieve an approximately high normal range of leptin level in those chimeras (33). For some experiments, CD4 T cells were isolated from SLE PBMCs, transfected with LepR expression vector or the control, and injected into the chimeras together with the CD4 T-depleted SLE PBMCs for immune reconstitution. Four weeks later, humanized chimeras were detected for splenic Tfh cells, serum IgG autoantibody, and urine protein. Experiments were conducted in accordance to the ARRIVE guidelines and approved by the Institutional Review Board of Soochow University.

### IgG anti-dsDNA and urine protein

Serum IgG anti-dsDNA and urine protein were analyzed as previously described (2, 34). Specifically, Human anti-dsDNA IgG ELISA Kit (Abnova) was used for IgG anti-dsDNA and Bradford Protein Assay Kit (GENEray) was used for urine protein analyses.

### Statistical analyses

All data were presented as mean ± SEM. The Mann-Whitney-Wilcoxon (MWW) test was used for two-group comparisons, and one-way ANOVA plus Tukey’s method was used for comparison of more than two groups. Spearman correlation analysis was used for testing correlations. All statistics were performed using GraphPad PRISM 9.0 (GraphPad Software Inc.) and p < 0.05 was considered significant.

## Results

### Circulating leptin inversely associates with disease activity in SLE patients

**Figure 1:**
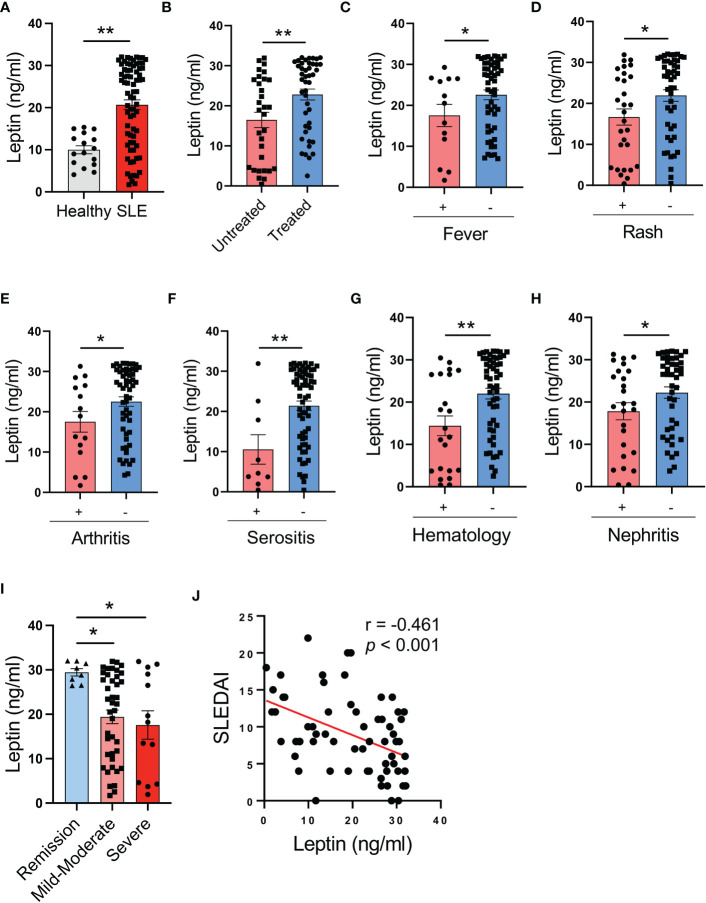
Circulating leptin is negatively associated with SLE disease activity. **(A)** Serological leptin in SLE patients (n=73) and healthy donors (n=16) was detected by ELISA. Each dot represents data from one individual. **p < 0.01 with unpaired MWW test. **(B)** SLE patients with (n=44) or without (n=31) treatment were analyzed for serum leptin levels. Each dot represents data from one individual. **p < 0.01 with unpaired MWW test. **(C–G)** SLE patients with or without the indicated manifestations were analyzed for serum leptin levels. Each dot represents data from one individual. *p < 0.05, **p < 0.01 with unpaired MWW test. **(H)** SLE patients with (n=26) or without (n=44) active nephritis was analyzed for serum leptin. Each dot represents data from one individual. *p < 0.05 with unpaired MWW test. **(I)** SLE patients with the indicated disease severity including remission (SLEDAI 0-4), mild-moderate (SLEDAI 5-14) and severe (SLEDAI >=15), were analyzed for serum leptin levels. Each dot represents data from one individual. *p < 0.05 with ANOVA plus Tukey’s method. **(J)** Serum leptin levels were inversely correlated with SLEDAI of SLE patients (n=65). Each dot represents data from one individual. Spearman correlation analyses.

To understand the potential role of leptin in human SLE, patients with SLE and healthy individuals were analyzed for serological levels of leptin. We found significantly higher levels of serum leptin in SLE patients (**Figure 1A**). Of note, patients under treatment showed further elevated levels of serum leptin than untreated patients (**Figure 1B**).

To determine the association of serum leptin with the disease activity of SLE patients, patients with or without the involvement of multiple manifestations and organs were analyzed for serum leptin. SLE patients without fever, rash, arthritis, serositis, hematologic disorders, and active nephritis exerted higher serum leptin levels (**Figures 1C–H**). In support, patients with severe disease (SLEDAI >=15) showed lower levels of serum leptin, while those with mild disease had higher serum leptin (**Figure 1I**). Finally, serum levels of leptin were inversely correlated with the disease activity of SLE patients (**Figure 1J**). Together, SLE patients exert higher levels of serum leptin, inversely associating with disease activity.

### Serum leptin predicts decreased IgG anti-dsDNA in SLE patients

**Figure 2:**
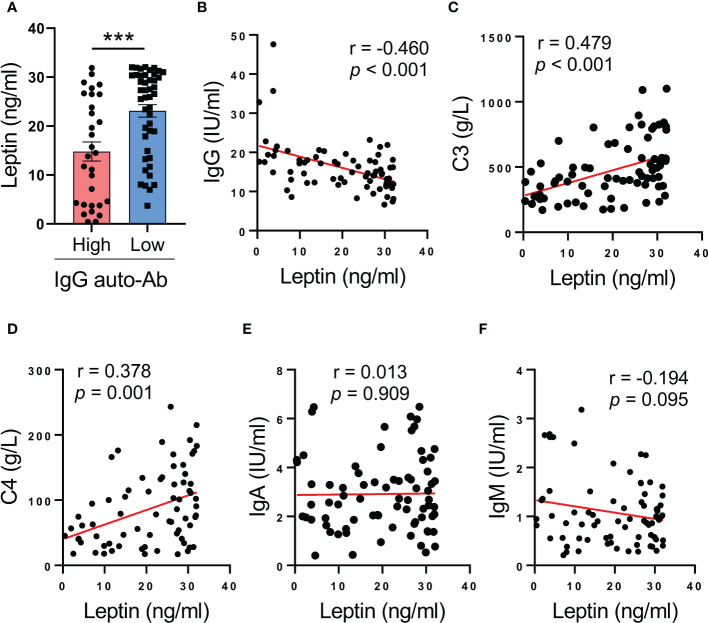
Higher serum leptin predicts impaired IgG anti-dsDNA generation in human SLE. **(A)** SLE patients with the higher (n=30) and lower (n=45) IgG anti-dsDNA was analyzed for serum leptin. Each dot represents data from one individual. ***p < 0.001 with unpaired MWW test. **(B)** Serum leptin levels were inversely correlated with IgG anti-dsDNA generation in SLE patients (n=71). Each dot represents data from one individual. Spearman correlation analyses. **(C, D)** Serum leptin levels were positively correlated with C3 and C4 levels in SLE patients. Each dot represents data from one individual. Spearman correlation analyses. **(E, F)** Serum leptin levels exerted no significant associations with IgA and IgM generations in SLE patients. Each dot represents data from one individual. Spearman correlation analyses.

To understand the inverse correlation between serum leptin and disease activity of SLE patients, serum leptin was analyzed for the association with serum levels of IgG anti-dsDNA autoantibody, the major driver for lupus onset and flares in human SLE. We observed that patients with lower levels of IgG anti-dsDNA exerted higher levels of serum leptin (**Figure 2A**). In support, serum leptin was negatively correlated with serum levels of IgG anti-dsDNA in SLE patients (**Figure 2B**). Meanwhile, serum leptin was also positively correlated with serum levels of C3 and C4 levels in human SLE (**Figures 2C, D**). The inverse correlation between serum leptin and IgG autoantibody was a selective phenomenon as serum leptin showed no significant correlations with IgA and IgM generations in SLE patients (**Figures 2E, F**).

### Leptin drives AMPK activation and inhibits differentiation of healthy Tfh cells

**Figure 3:**
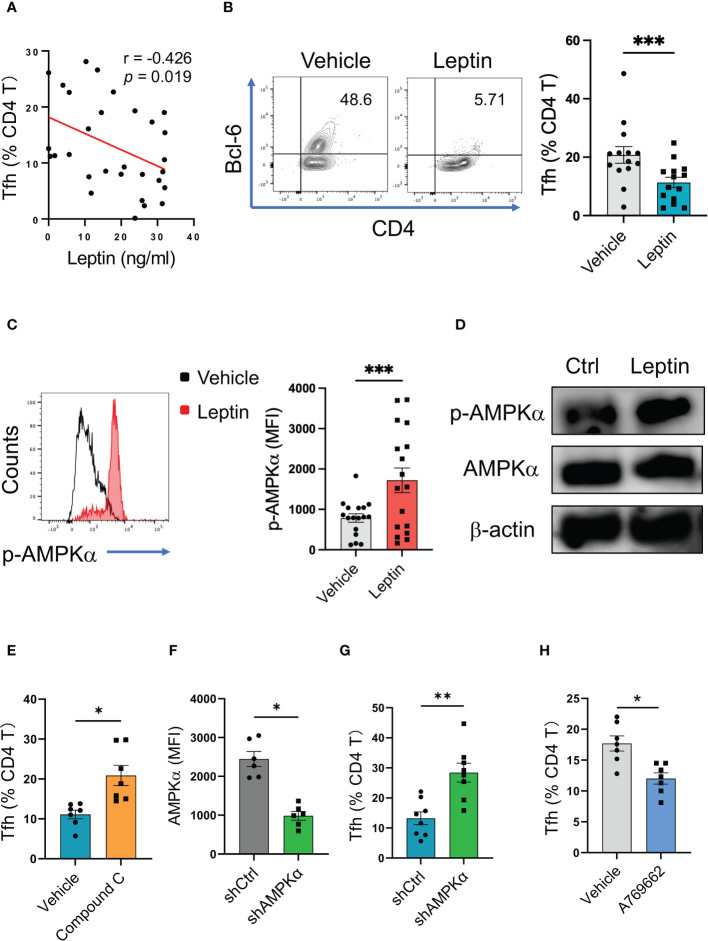
Leptin inhibits human Tfh cell differentiation *via* AMPK. **(A)** Serum leptin levels were associated with circulating Bcl-6-expressing Tfh cell frequencies in SLE patients (n=30). Each dot represents data from one individual. Spearman correlation analyses. **(B)** CD4 T cells from healthy donors (n=14) were stimulated with anti-CD3/CD28 beads in the presence or absence of recombinant leptin protein (100 ng/ml) for 4 days, and analyzed for Bcl-6-expressiong Tfh cells. Each dot represents data from one individual. ***p < 0.001 with paired MWW test. **(C)** CD4 T cells from healthy donors (n=17) were stimulated with anti-CD3/CD28 beads in the presence or absence of recombinant leptin protein (100 ng/ml) for 3 days, and analyzed for intracellular phosphor-AMPKα using PhosFlow. Each dot represents data from one individual. ***p < 0.001 with paired MWW test. **(D)** Healthy CD4 T cells were stimulated with anti-CD3/CD28 beads in the presence or absence of recombinant leptin protein (100 ng/ml) for 3 days, and analyzed for intracellular phosphor-AMPKα using immunoblots. A representative from 4 donors. **(E)** CD4 T cells from healthy donors (n=7) were stimulated with anti-CD3/CD28 beads plus recombinant leptin protein (100 ng/ml), together with or without Compound C (10 μM), for 4 days, and analyzed for Bcl-6-expressiong Tfh cells. Each dot represents data from one individual. *p < 0.05 with paired MWW test. **(F)** CD4 T cells from healthy donors (n=6) were transfected with AMPKα shRNA or the control, stimulated with anti-CD3/CD28 beads for 24 hrs, and analyzed for intracellular AMPKα levels using flow cytometry. Each dot represents data from one individual. *p < 0.05 with paired MWW test. **(G)** CD4 T cells from healthy donors (n=8) were transfected with AMPKα shRNA or the control, and stimulated with anti-CD3/CD28 beads for 4 days. Each dot represents data from one individual. **p < 0.01 with paired MWW test. **(H)** CD4 T cells from healthy donors (n=7) were stimulated with anti-CD3/CD28 beads in the presence or absence of A769662 (10 μM) for 4 days. Each dot represents data from one individual. *p < 0.05 with paired MWW test.

To detect why leptin was negatively associated with IgG autoantibody in SLE patients, we detected the relationship between leptin and Tfh cells, a CD4 T cell subset that licenses IgG autoantibody generation (35). In line with the inverse correlation of serum leptin with IgG anti-dsDNA, serum leptin levels were negatively associated with circulating frequencies of Bcl-6-expressing Tfh cells in SLE patients (**Figure 3A**).

To evaluate the effect of leptin on Tfh cell differentiation, CD4 T cells from healthy donors were activated with anti-CD3/CD28 beads in the presence or absence of leptin. Such a cytokine-unbiased activation of human CD4 T cells represents a cell-intrinsic and spontaneous differentiation of T cells. Administration of leptin inhibited the differentiation of Tfh cells from healthy CD4 T cells (**Figures 3B**; **S1A**), assigning leptin as an inhibitory player for human Tfh cell differentiation.

To explore how leptin inhibited the differentiation of human Tfh cells, CD4 T cells from healthy donors were activated with anti-CD3/CD28 beads plus leptin and analyzed for intracellular phosphor-AMPK. As a result, leptin efficiently promoted the activation of AMPK, leading to increased levels of p-AMPK in healthy CD4 T cells (**Figure 3C**). Such a phenomenon was further confirmed with immunoblots, showing robust levels of p-AMPK in response to leptin in healthy T cells (**Figure 3D**).

To determine whether leptin suppressed Tfh cell differentiation in an AMPK-dependent manner, CD4 T cells from healthy donors were activated with anti-CD3/CD28 beads, leptin, and Compound C, a specific AMPK inhibitor. Blockade of AMPK by Compound C abrogated leptin-mediated inhibition of Tfh cell differentiation, resulting in increased frequencies of Tfh cells (**Figures 3E**; **S1B**). These data demonstrate a crucial function of AMPK activation in impaired Tfh cell differentiation upon leptin treatment. In support, the genetic knockdown of AMPKα also abrogated the suppressive function of leptin in Tfh cell differentiation (**Figures 3F, G**). In line with the unfavorable function of AMPK in Tfh cell differentiation, activation of AMPK by A769662 reduced the differentiation of Tfh cells from healthy CD4 T cells (**Figure 3H**). Thus, leptin activates AMPK for inhibiting the differentiation of healthy Tfh cells.

### Leptin fails to reduce Tfh cells in human SLE

**Figure 4:**
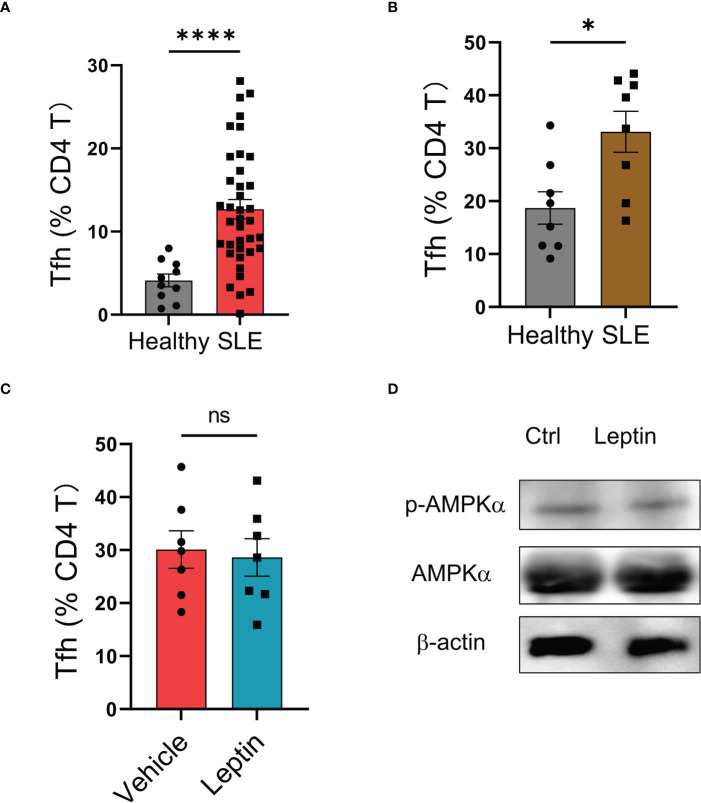
Leptin exerts no significant effect on SLE Tfh cells. **(A)** SLE patients (n=38) and healthy donors (n=10) were analyzed for circulating frequencies of Bcl-6-expressing Tfh cells. Each dot represents data from one individual. ****p < 0.0001 with unpaired MWW test. **(B)** CD4 T cells from SLE patients (n=8) or healthy controls (n=8) were stimulated with anti-CD3/CD28 beads for 4 days. Each dot represents data from one individual. *p < 0.05 with unpaired MWW test. **(C)** CD4 T cells from SLE patients (n=7) were stimulated with anti-CD3/CD28 beads in the presence or absence of recombinant leptin protein (100 ng/ml) for 4 days. Each dot represents data from one individual. Unpaired MWW test. **(D)** SLE CD4 T cells were stimulated with anti-CD3/CD28 beads in the presence or absence of recombinant leptin protein (100 ng/ml) for 3 days and analyzed for intracellular phosphor-AMPKα using immunoblots. A representative from 4 patients. ns, no significance.

Although leptin exerted an inhibitory function in human Tfh cell differentiation (**Figure 3**), the circulating frequency of Bcl-6-expressing Tfh cells in SLE patients was still higher than that in healthy controls (**Figure 4A**), suggesting an impaired function of leptin in SLE CD4 T cells. In support, we found a spontaneous mal-differentiation of Tfh cells of SLE CD4 T cells, showing an elevated frequency of Bcl-6-expressing CD4 T cells upon cytokine-unbiased anti-CD3/CD28 activation (**Figures 4B**; **S1C**). The coexistence of higher serum leptin and circulating Tfh cells suggest that leptin was still unable to block Tfh cell differentiation in clinical patients. Indeed, administration of leptin protein exerted a minimum effect on the mal-differentiation of SLE CD4 T cells (**Figure 4C**). In consistency, leptin failed to promote the activation of AMPK in CD4 T cells from SLE patients (**Figure 4D**).

### The deficiency of LepR licenses Tfh cell differentiation in SLE patients

**Figure 5:**
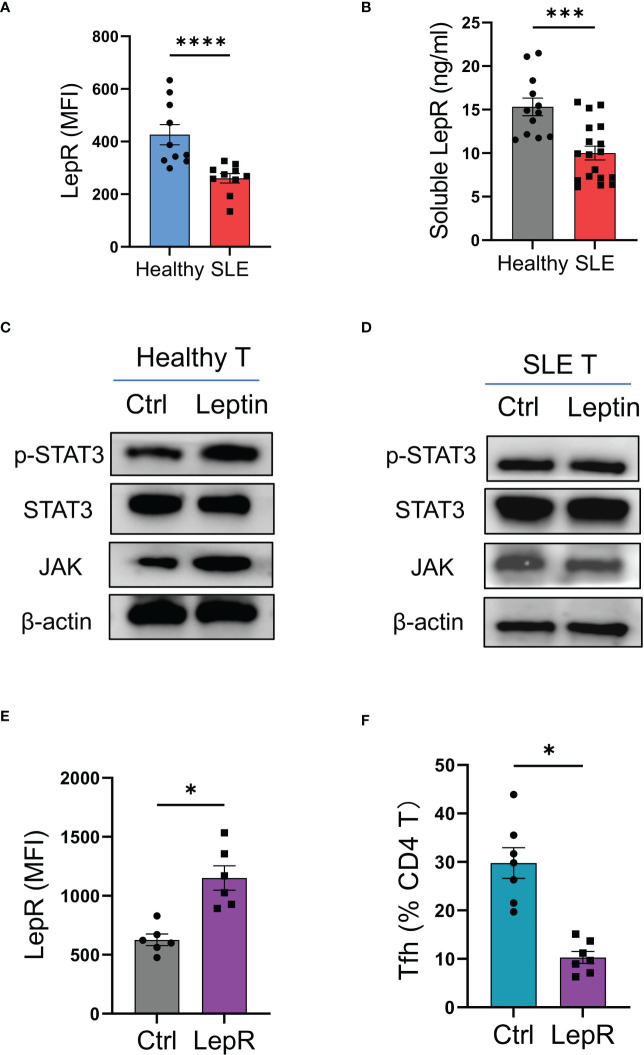
LepR deficiency blocks leptin-mediated inhibition on SLE Tfh cells. **(A)** Circulating CD4 T cells from SLE patients (n=10) and healthy donors (n=10) were detected for LepR expressions using flow cytometry. Each dot represents data from one individual. ****p < 0.0001 with unpaired MWW test. **(B)** Serum levels of soluble LepR in SLE patients (n=18) and healthy controls (n=12) were detected by ELISA. Each dot represents data from one individual. ***p < 0.001 with unpaired MWW test. **(C, D)** CD4 T cells from healthy donors and SLE patients were stimulated with or without recombinant leptin protein (100 ng/ml) for 1 hour and analyzed for intracellular phosphor-Stat3, total Stat3, and JAK using immunoblots. Representatives from 4 patient-healthy pairs. **(E)** CD4 T cells from SLE patients (n=6) were transfected with LepR expression vector or the control, and stimulated with anti-CD3/CD28 beads for 24 hrs, followed by analyses for LepR expressions using flow cytometry. Each dot represents data from one individual. *p < 0.05 with paired MWW test. **(F)** CD4 T cells from SLE patients (n=7) were transfected with LepR expression vector or the control and stimulated with anti-CD3/CD28 beads plus recombinant leptin protein (100 ng/ml) for 4 days. Each dot represents data from one individual. *p < 0.05 with paired MWW test.

To explore why leptin could not efficiently inhibit the mal-differentiation of Tfh cells in SLE, CD4 T cells from SLE patients and healthy donors were analyzed for expressions of LepR. We found that LepR expression was significantly lower in SLE CD4 T cells than that in healthy controls (**Figure 5A**). In addition, serum levels of soluble LepR were also decreased in SLE patients (**Figure 5B**).

To detect the LepR signaling, healthy and SLE T cells were treated with leptin respectively, and detected for intracellular phosphor-Stat3 and JAK levels. We found that leptin efficiently induced the phosphorylation of Stat3 and increased the level of JAK in healthy T cells, but did not exert such a significant effect in SLE T cells (**Figures 5C, D**).

To test whether LepR deficiency was responsible for the impaired function of leptin in SLE, CD4 T cells from SLE patients were transfected with LepR expression vector and tested for Tfh cell differentiation under leptin treatment. Overexpression of LepR in SLE CD4 T cells rescued the inhibitory effect of leptin on the mal-differentiation of SLE CD4 T cells, resulting in decreased Bcl-6-expressing Tfh cells (**Figures 5E, F**). Those data pinpoint LepR deficiency as a cell-intrinsic basis for the compromised effect of leptin on Tfh cell differentiation in human SLE.

### Targeting LepR in CD4 T cells protects from Tfh cell mal-differentiation and auto-IgG generation in humanized lupus chimeras

**Figure 6:**
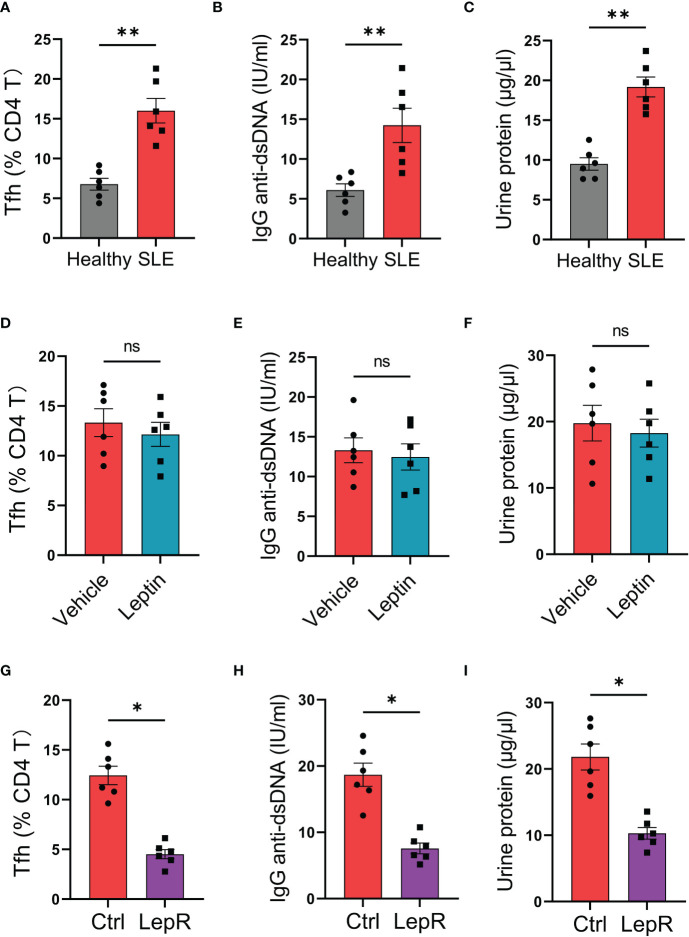
Targeting LepR recuses inhibitory activities of leptin on lupus development in humanized chimeras. **(A–C)** NSG mice reconstituted with SLE PBMCs (n=6) and healthy PBMCs (n=6) respectively were detected for splenic Bcl-6-expressing Tfh cells, serum IgG anti-dsDNA and urine protein at 4^th^ week. Each dot represents data from one chimera. **p < 0.01 with unpaired MWW test. **(D–F)** Humanized SLE chimeras were treated with (n=6) or without (n=6) low-dose recombinant leptin protein after immune reconstitution, and analyzed for splenic Bcl-6-expressing Tfh cells, serum IgG anti-dsDNA plus urine protein at 4^th^ week. Each dot represents data from one chimera. Paired MWW test. **(G–I)** SLE CD4 T cells were transected with LepR expression vector (n=6) or the control (n=6) prior to immune reconstitution of chimeras. Splenic Bcl-6-expressing Tfh cells, serum IgG anti-dsDNA and urine protein were analyzed at 4^th^ week post immune reconstitution plus low-dose leptin treatment. Each dot represents data from one chimera. *p < 0.05 with paired MWW test. ns, no significance.

To test the translational application of LepR-based therapeutic strategy for SLE treatment, we established humanized lupus chimeras in which immune-deficient NSG mice were reconstituted with PBMCs from active lupus patients (2). Those patients’ immune cells resulted in higher frequencies of Tfh cells in the spleen of chimeras, accompanied by elevated levels of serum IgG anti-dsDNA (**Figures 6A, B**). Besides, such mal-differentiation of Tfh cells and IgG generations from SLE immune cells were pathogenic, leading to robust levels of urine protein in humanized lupus chimeras (**Figure 6C**). Meanwhile, administration of recombinant leptin protein exerted a minimum effect on the Tfh cell differentiation, IgG anti-dsDNA generation and urine protein of those chimeras (**Figures 6D–F**).

Using the humanized lupus model, we tested whether targeting LepR by genetic overexpression could protect the chimeras from Tfh cell mal-differentiation and IgG anti-dsDNA generation. Specifically, CD4 T cells were isolated from the patient’s PBMCs, transfected with LepR expression vector, and placed back into the PBMCs for humanized immune reconstitution of NSG mice. We found that overexpression of LepR in CD4 T cells enhanced the inhibitory function of leptin in controlling SLE development (**Figures 6G–I**), leading to decreased Tfh cells, impaired IgG anti-dsDNA generation, and reduced urine protein. In collective, targeting LepR is a promising strategy for inhibiting the disease progression of human SLE.

## Discussion

Tfh cells are crucially involved in autoimmunity by supporting the generation of IgG autoantibodies (36). While metabolic adaption is well-acknowledged to be the determinant for T cell differentiation and function, underpinning mechanisms for Tfh cell mal-differentiation in human SLE remain unclear. In this study, we identify leptin as an inhibitory factor for human Tfh cell differentiation, negatively associated with the disease activity of SLE patients. Of interest, deficiency of LepR abrogates the leptin-mediated inhibition of SLE Tfh cell differentiation, resulting in the coexistence of high circulating leptin and increased Tfh cells in those patients. Targeting LepR in CD4 T cells efficiently restores the inhibitory effect of leptin on Tfh cell differentiation and IgG anti-dsDNA response, serving as a promising therapeutic strategy for human SLE.

As an approved drug, leptin is produced predominantly from adipose tissue (37), and functions as an afferent signal in a negative feedback loop that maintains homeostatic control of adipose tissue mass (38). In this study, we confirmed an elevated level of circulating leptin in patients with SLE, indicating an increased adipose tissue in those patients. In support, the BMI of SLE patients was positively associated with circulating leptin levels and negatively correlated with the SLEDAI (**Figure S2**). In consistence, SLE patients have been reported to have increased adiposity distribution than healthy controls, including the volume and mass of visceral adipose tissue, contributing to disease development as a cardiovascular risk factor (39, 40). While corticosteroids are widely used in treatment of SLE patients, such treatment frequently drives adiposity and the leptin secretion (41, 42). In lupus-prone NZB/WF1 mice, low fiber intake also deteriorates disease progression as reflected by accelerated mortality and autoantibody production (43). Mechanistically, an increase in white adipose tissue mass and fat inflammation goes along with systemic, low-grade inflammation driving autoimmunity in those low fiber-fed lupus-prone mice (43). Thus, circulating leptin interlinks obesity and inflammation, serving as a critical marker for inflammatory immune-mediated conditions including SLE.

Encoded by the LEPR gene, LepR has six isoforms including LepRa-f, which share a common leptin binding domain but differ in intracellular domains (37, 44). While LepRa-d and f are trans-membrane receptors that recruit JAK2, LepRe uniquely lacks a transmembrane domain and is a soluble isoform (37). Of note, LepRb features an extended intracellular domain that can be phosphorylated by JAK2 at Y985, Y1077and Y1138, accounting for predominant functions of leptin in energy homeostasis (37, 45). Typically, Y985 activates SHP-2 and MAPK signaling, Y1077 activates STAT5 signaling and Y1138 activates STAT3 signaling (37, 45). As for energy metabolism, leptin induces mTOR signaling and inhibits AMPK activity to control food intake *via* the regulation of hypothalamic neuropeptides (37, 46). However, leptin drives AMPK activation in the peripheral to promote catabolic pathways (37, 47). Herein, we identify LepR deficiency in CD4 T cells of SLE patients, along with the decreased level of soluble LepR in the circulation of those patients, indicating a possible mutation of the encoding LEPR gene in human SLE. In fact, LepR deficiency is an autosomal-recessive endocrine disorder that is currently underdiagnosed because of the lack of access to genetic testing and insufficient recognition (48). For SLE patients, the expression of LepR was generally comparable in patients with active disease and those in remission (**Figure S3**), assigning LepR deficiency in CD4 T cells as an inflammation-induced phenomenon unlikely.

While previous studies mainly focus on LepRb-expressing neurons and reveal the hypothalamus as the main target for central leptin activity, here we identify the stimulatory effect of leptin on the AMPK activity of human CD4 T cells. Meanwhile, leptin deficiency could impair the maturation of dendritic cells and enhance the induction of regulatory T and Th17 cells (49), indicating an inhibitory effect of leptin on Treg and Th17 cells. In contrast, leptin might also directly promote T-cell glycolytic metabolism to drive effector T-cells in a mouse model of autoimmunity (50), indicating a complicated function of leptin T cell biology. Herein, we demonstrated an enhanced AMPK activity of human CD4 T cells in response to leptin under cytokine-unbiased conditions. Of note, we uncover the deficiency of LepR as a cell-intrinsic feature for CD4 T cells from SLE patients, impairing leptin activity in peripheral and resulting in Tfh cell mal-differentiation with disease progression. Consequently, higher serum leptin coexists with increased circulating Tfh cells and IgG anti-dsDNA autoantibody in SLE patients. Of importance, LepR is therapeutically targetable as overexpression of LepR rescues the inhibitory effect of leptin on SLE Tfh cell differentiation, and blocks IgG autoantibody production in humanized lupus chimeras. However, we would like to admit some limitations of this study. One is that current findings were obtained in a subset of clinical patients and a relatively large patient cohort would be beneficial to support our findings. Of note, one report showed an advancing effect of leptin on human Tfh cells (51). Specifically, leptin might promote the differentiation of human Tfh cells under cytokine-biased conditions, indicating complex signaling interactions between LepR and the Tfh-related cytokines including IL-12, IL-23, and TFG-β (51). Another is that we are not sure whether LepR deficiency is a selective phenomenon for SLE in autoimmune settings, which relies on other patient cohorts. The third is that while SLE patients are reported to have increased adipose tissue (39, 40), which could be a consequence of clinical treatment using glucocorticoid, precise molecular mechanisms underlying leptin production and LepR deficiency in SLE patients remain unclear.

In summary, Leptin-LepR signaling appears to be defective in some SLE patients. Specifically, leptin can activate AMPK in human CD4 T cells and thus inhibit Tfh cell differentiation. However, LepR deficiency acts as an intrinsic feature for SLE CD4 T cells, blocking the leptin-mediated inhibitory effects. Accordingly, targeting LepR by genetic overexpression is efficient in restoring leptin function and alleviating disease progression in humanized SLE chimeras. Such findings were obtained from SLE patients, reflecting realistic conditions in clinical practice and assigning LepR as an opportunistic therapeutic target for SLE management.

## Data availability statement

The original contributions presented in the study are included in the article/**Supplementary Material**. Further inquiries can be directed to the corresponding authors.

## Ethics statement

The studies involving human participants were reviewed and approved by Ethics Committee of Soochow University and Ethics Committee of Nanjing Medical University. The patients/participants provided their written informed consent to participate in this study. The animal study was reviewed and approved by Ethics Committee of Soochow University and Ethics Committee of Nanjing Medical University.

## Author contributions

ZW, FY, and MJZ designed the study. TL, LJ, and MGZ collected samples, performed experiments, and analyzed data. MW and LT participated in experimental design and data analyses. ZW wrote the manuscript with input from all authors. All authors contributed to the article and approved the submitted version.
