# Supplementary material for: Deficient leptin receptor signaling in T cells of human SLE
Source: Front Immunol. 2023 Mar 17;14:1157731. doi: 10.3389/fimmu.2023.1157731 (PMC10063787; doi:10.3389/fimmu.2023.1157731)

Unedited gel for Fig.3D

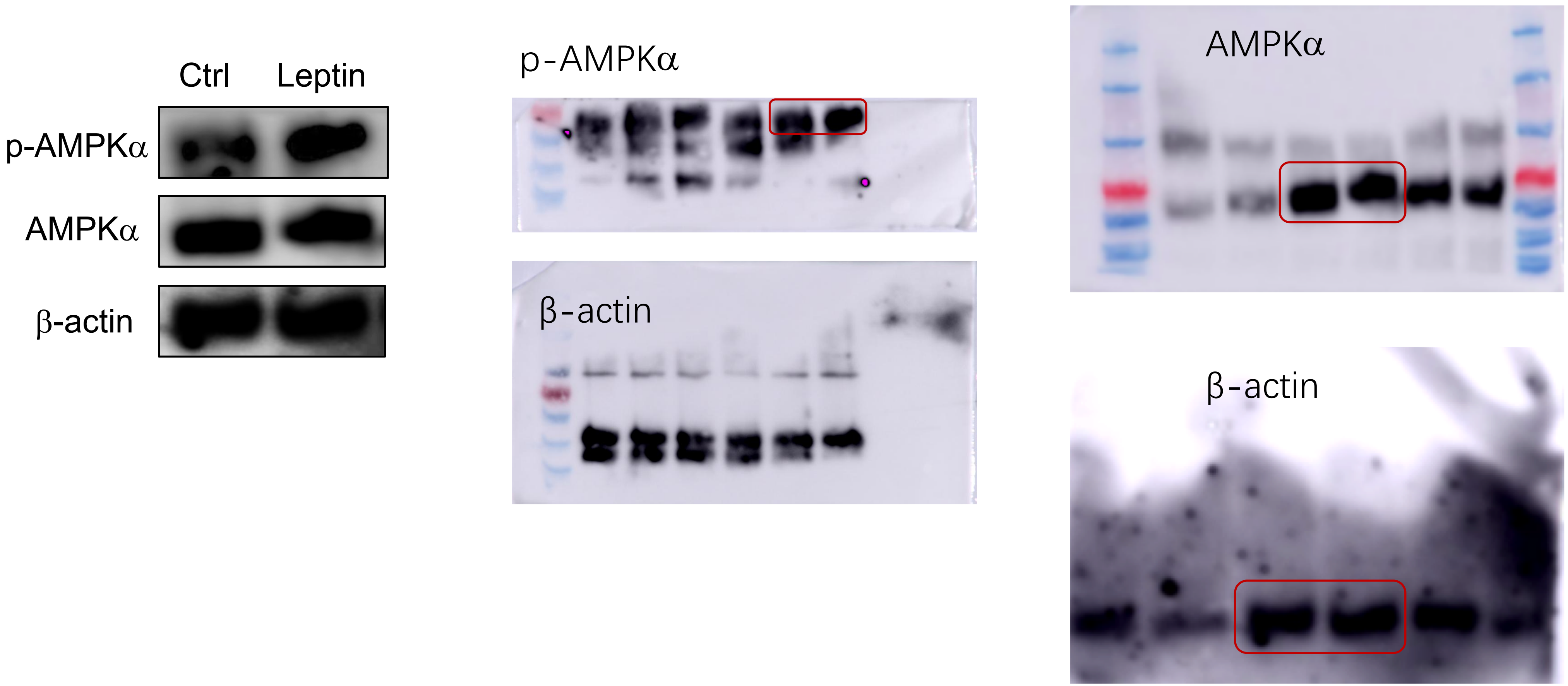

Unedited gel for Fig.4D

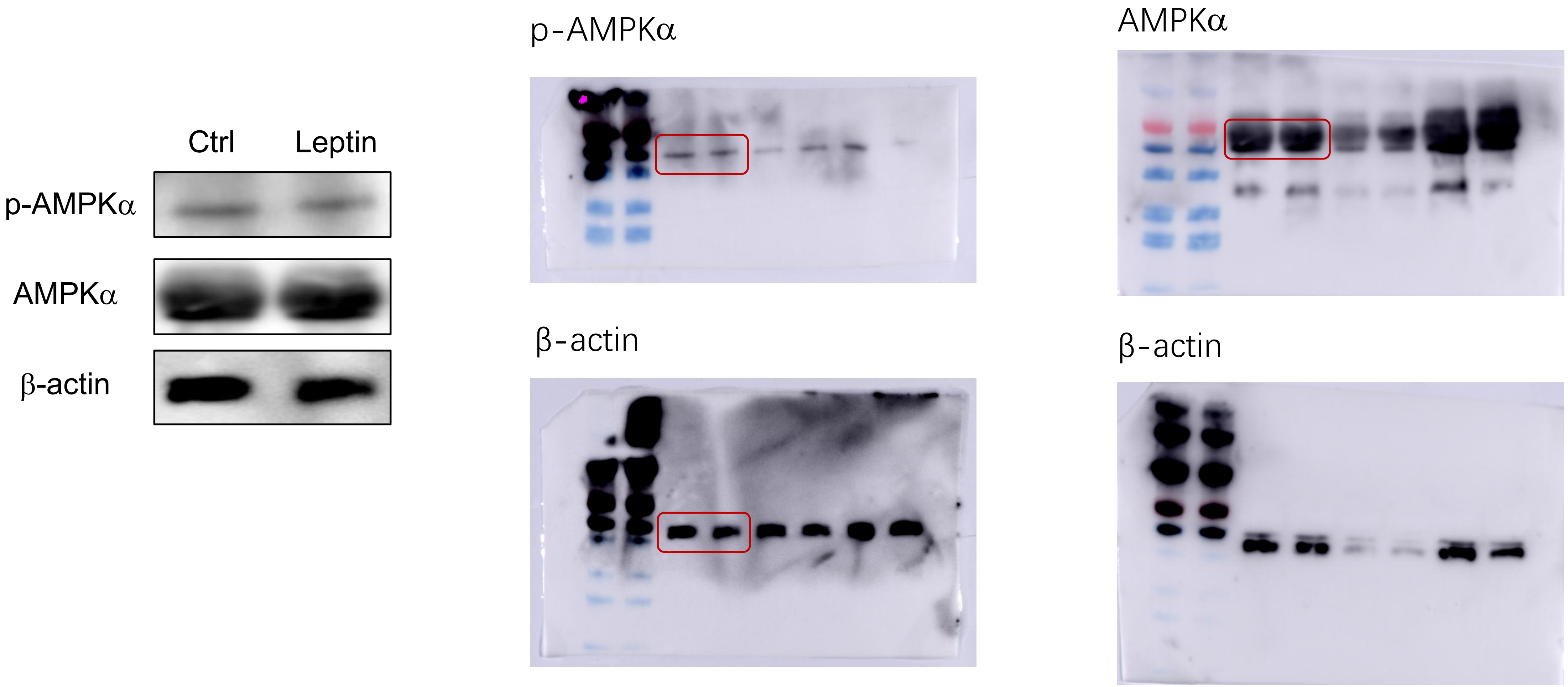

Unedited gel for Fig.5C

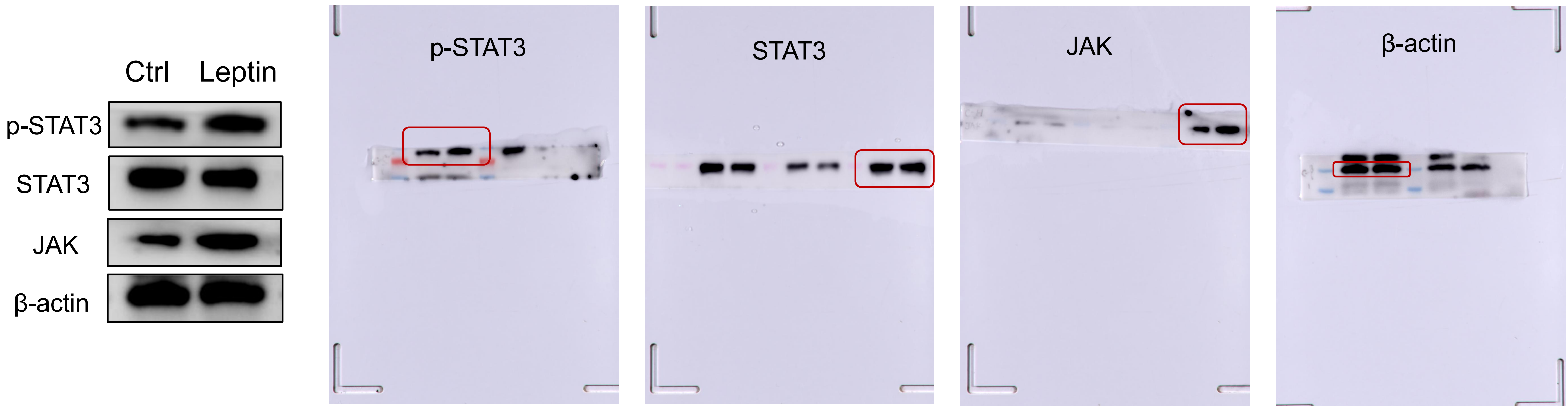

Unedited gel for Fig.5D

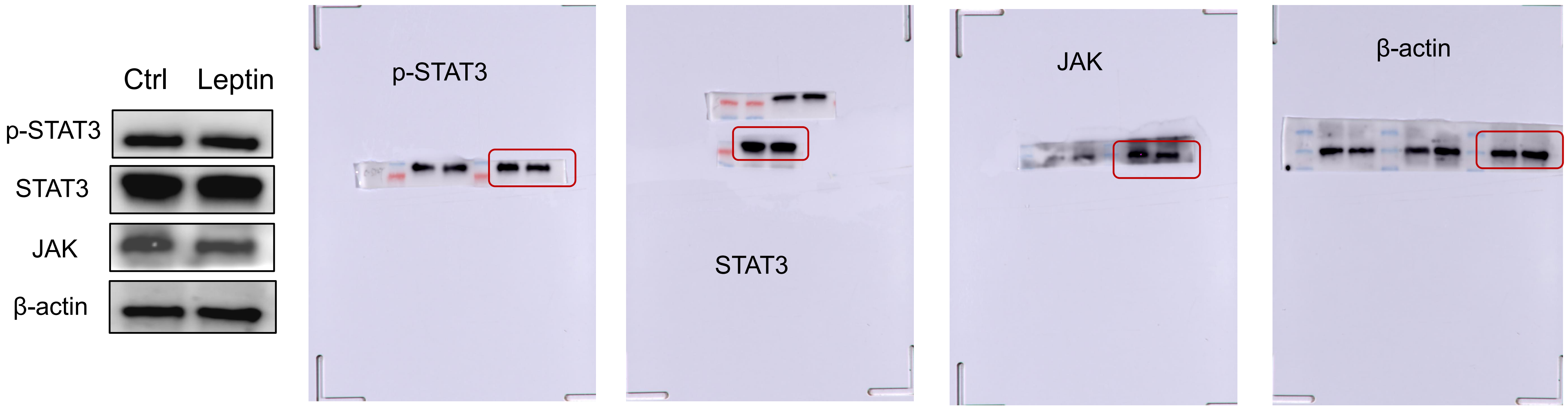

Gating strategy for Fig.3B: purified CD4 T cells

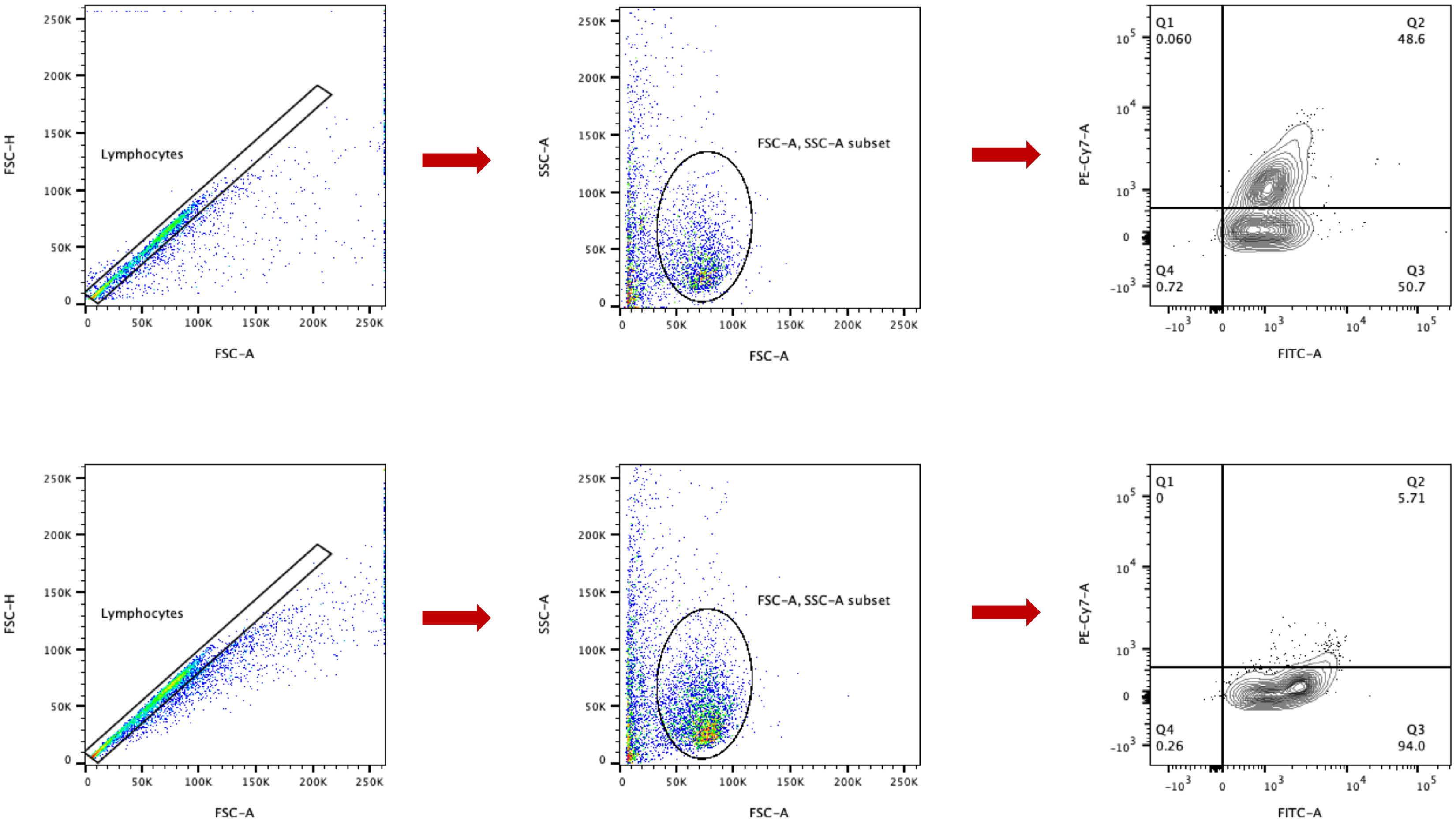

Gating strategy for Fig.3C: purified CD4 T cells

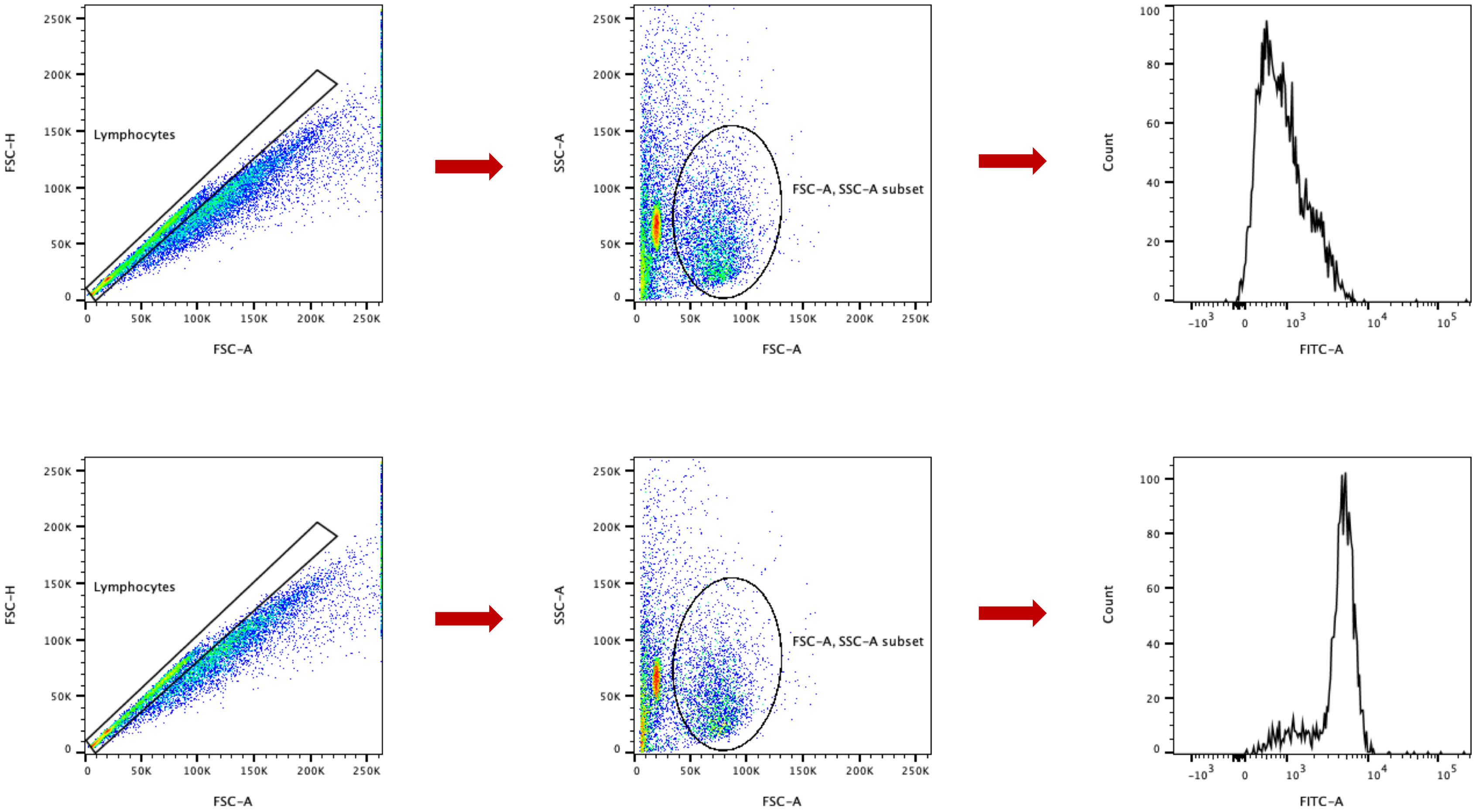

Supplement: Supplementary file 1 [file DataSheet_1.pdf]
