# Supplementary figures and images for: Deficient leptin receptor signaling in T cells of human SLE
Source: Front Immunol. 2023 Mar 17;14:1157731. doi: 10.3389/fimmu.2023.1157731 (PMC10063787; doi:10.3389/fimmu.2023.1157731)

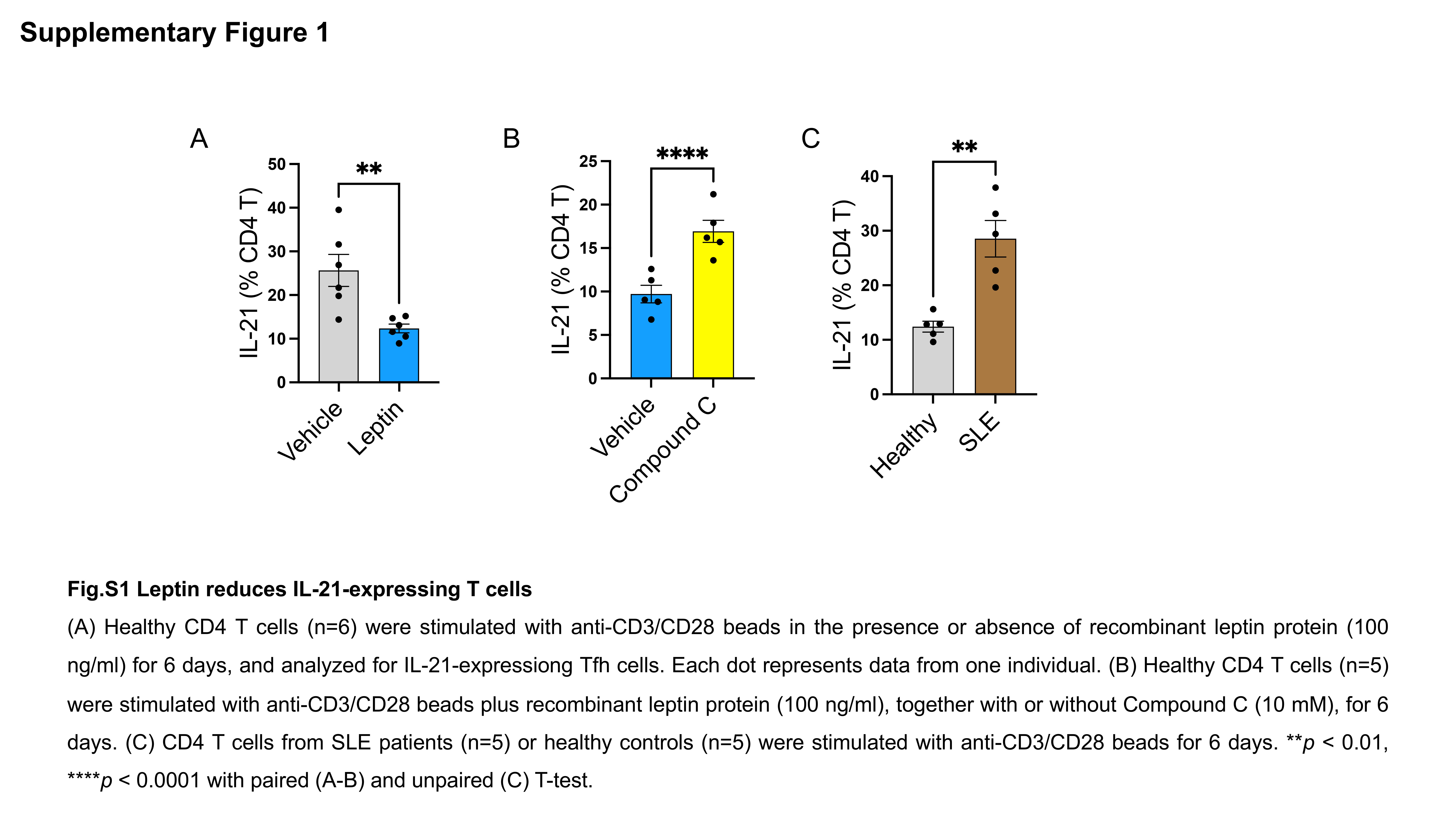

Supplement: Supplementary file 2 [file Image_1.jpeg]

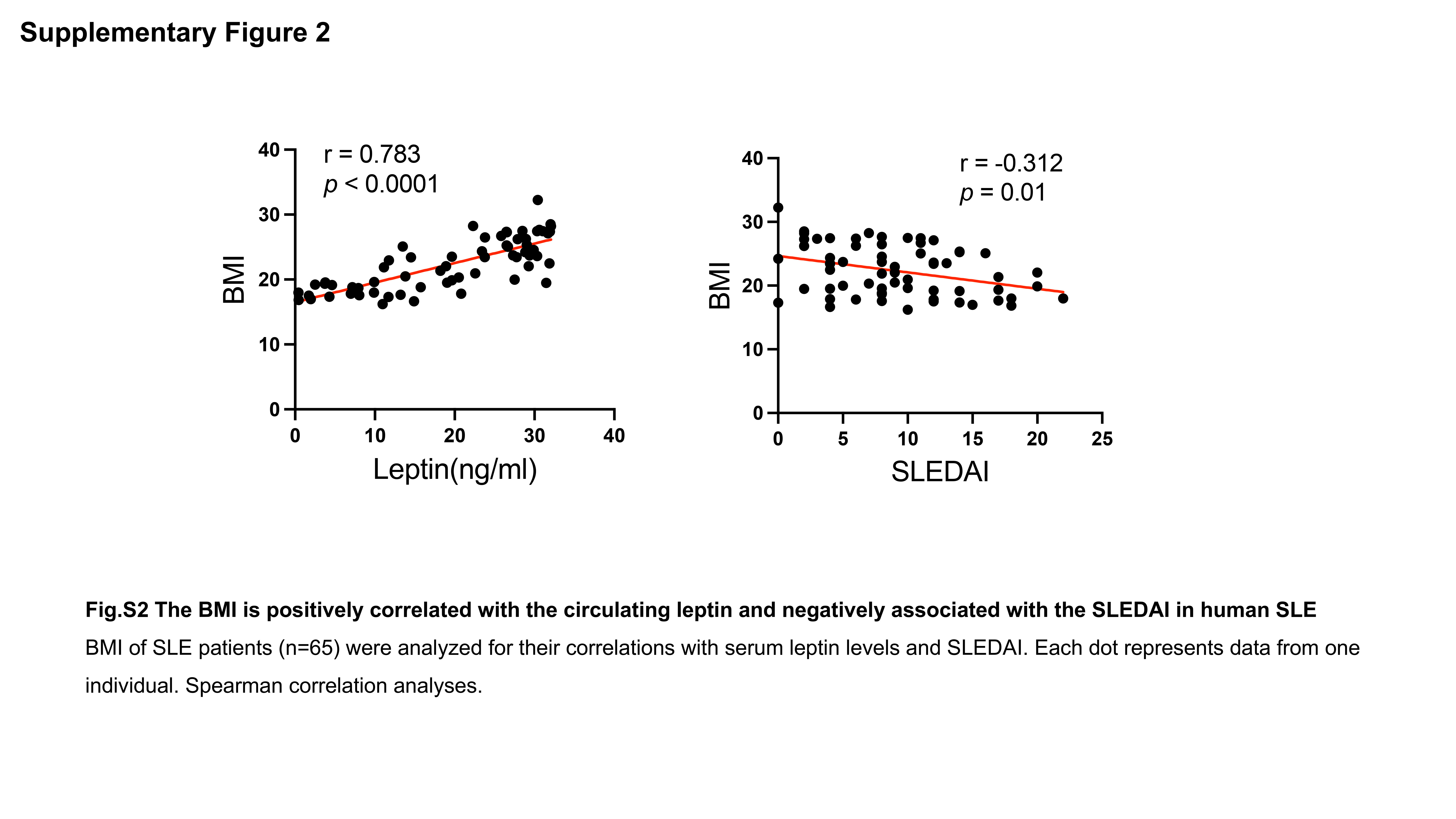

Supplement: Supplementary file 3 [file Image_2.jpeg]

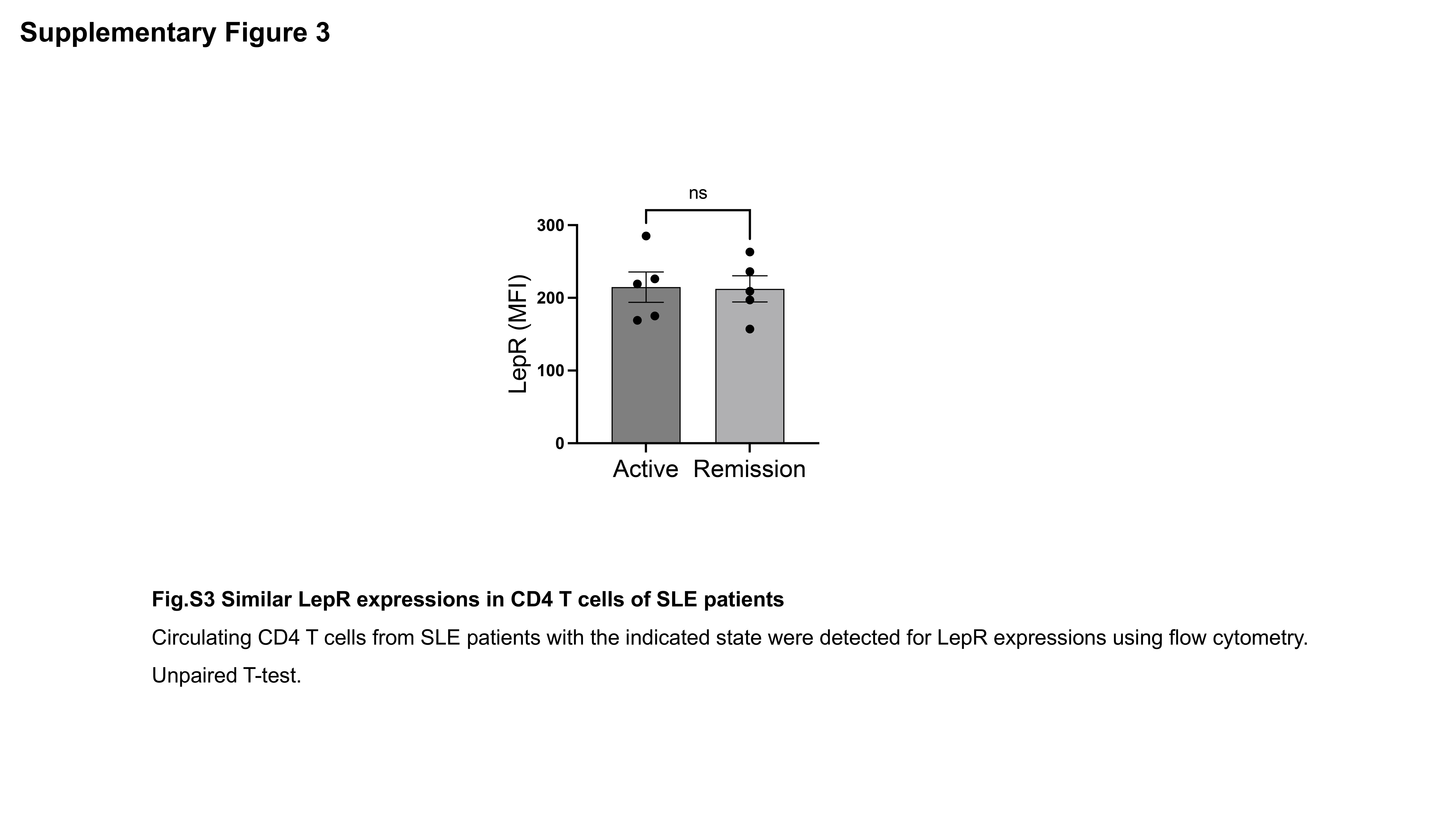

Supplement: Supplementary file 4 [file Image_3.jpeg]
